# Evaluation of Drought Responses in Two *Tropaeolum* Species Used in Landscaping through Morphological and Biochemical Markers

**DOI:** 10.3390/life13040960

**Published:** 2023-04-06

**Authors:** Diana M. Mircea, Roberta Calone, Rashmi Shakya, Javier Zuzunaga-Rosas, Radu E. Sestras, Monica Boscaiu, Adriana F. Sestras, Oscar Vicente

**Affiliations:** 1 Department of Forestry, University of Agricultural Sciences and Veterinary Medicine Cluj-Napoca, 3-5 Manastur Street, 400372 Cluj-Napoca, Romania; diana-maria.mircea@usamvcluj.ro; 2 Institute for the Conservation and Improvement of Valencian Agrodiversity (COMAV), Universitat Politècnica de València, Camino de Vera s/n, 46022 Valencia, Spain; rashmi.shakya@mirandahouse.ac.in (R.S.); ovicente@upvnet.upv.es (O.V.); 3 CREA—Council for Agricultural Research and Economics, Research Centre for Agriculture and Environment, I-40128 Bologna, I-00184 Rome, Italy; roberta.calone@crea.gov.it; 4 Department of Botany, Miranda House, University of Delhi, Delhi 110007, India; 5 Department of Plant Production, Universitat Politècnica de València, Camino de Vera s/n, 46022 Valencia, Spain; jazuro@doctor.upv.es; 6 Department of Horticulture and Landscape, University of Agricultural Sciences and Veterinary Medicine Cluj-Napoca, 3-5 Manastur Street, 400372 Cluj-Napoca, Romania; rsestras@usamvcluj.ro; 7 Mediterranean Agroforestry Institute (IAM), Universitat Politècnica de València, Camino de Vera s/n, 46022 Valencia, Spain

**Keywords:** abiotic stress, optical sensor, ornamental plants, reactive oxygen species, spectrophotometry

## Abstract

One of the most important challenges horticultural crops confront is drought, particularly in regions such as the Mediterranean basin, where water supplies are usually limited and will become even scarcer due to global warming. Therefore, the selection and diversification of stress-tolerant cultivars are becoming priorities of contemporary ornamental horticulture. This study explored the impact of water stress on two *Tropaeolum* species frequently used in landscaping. Young plants obtained by seed germination were exposed to moderate water stress (half the water used in the control treatments) and severe water stress (complete withholding of irrigation) for 30 days. Plant responses to these stress treatments were evaluated by determining several growth parameters and biochemical stress markers. The latter were analysed by spectrophotometric methods and, in some cases, by non-destructive measurements using an optical sensor. The statistical analysis of the results indicated that although the stress responses were similar in these two closely related species, *T. minus* performed better under control and intermediate water stress conditions but was more susceptible to severe water stress. On the other hand, *T. majus* had a stronger potential for adaptation to soil water scarcity, which may be associated with its reported expansion and naturalisation in different regions of the world. The variations in proline and malondialdehyde concentrations were the most reliable biochemical indicators of water stress effects. The present study also showed a close relationship between the patterns of variation of flavonoid and chlorophyll contents obtained by sensor-based and spectrophotometric methods.

## 1. Introduction

One of the main challenges facing horticultural crops is drought, particularly in regions such as the Mediterranean basin, where water availability is frequently insufficient. Due to the increased water deficit brought about by global climatic changes, water is becoming a scarcer and more expensive resource and irrigation will be utilised more sparingly. Therefore, the selection and diversification of stress-tolerant cultivars are becoming more relevant in all horticultural fields, including floriculture [1,2]. Research on ornamental species’ responses to water stress and the mechanisms underlying these responses has significantly increased in recent decades [3,4,5,6,7,8,9].

Responses to water stress may vary between species or even cultivars of the same species, particularly in deficit irrigation situations when a genotype-dependent response is expected [10,11,12,13]. The conventional method for selecting drought-tolerant cultivars has entailed cultivating plants in water-stressed settings and comparing their growth and reproductive parameters to those recorded in plants in non-stressed environments. However, more recently, plant breeding efforts have switched to alternate methods of screening for stress tolerance, including the use of physiological [14,15,16,17] and biochemical markers [10,18,19,20,21]. Amongst the biochemical indicators of stress, the most widely used are those related to photosynthetic pigments, osmolytes, oxidative stress markers and antioxidants [4,22,23].

One of the main elements of the chloroplast, the relative chlorophyll content, is strongly correlated with the rate of photosynthetic activity [24,25]. The primary restriction on photosynthesis during mild to moderate drought is stomatal closure. To stop further water loss during a water shortage, plants close their stomata, which reduces the amount of CO_2_ available for photosynthesis and eventually results in a drop in nicotinamide adenine dinucleotide phosphate (NADPH). In addition to stomatal restrictions, leaf photochemical and biochemical deficiencies also contribute to a reduction in photosynthesis during water stress [24,25]. The gradual down-regulation or suppression of metabolic activities reduces RuBP content, which becomes the primary constraint during severe drought and prevents the photosynthetic uptake of CO_2_ [26]. As a result of chlorophyll degradation and pigment photo-oxidation under drought-stress circumstances, the decrease in chlorophyll concentration might be regarded as a typical sign of oxidative stress [27,28]. Drought promotes oxidative stress by reducing CO_2_ assimilation, which results in excess excitation energy and electron flow to O_2_ and uncontrolled generation of reactive oxygen species (ROS). ROS are by-products of plant aerobic metabolism [29], generated in various cellular compartments. They play a crucial role as signalling molecules involved in stress responses and plant growth, but when in excess may cause irreparable DNA damage and cell death [30]. In plants, ROS exist in ionic and/or molecular states. Each form of ROS has a distinctive oxidative capacity and influences different physiological and biochemical processes. Hydrogen peroxide (H_2_O_2_) is recognised as an important redox molecule because of its distinctive physical and chemical properties, including its outstanding stability inside cells and the fast and reversible oxidation of target proteins [31]. H_2_O_2_ is involved in cell signalling control, cell differentiation, senescence, and cell wall construction in plants. However, when in excess, it leads to oxidative damage [32,33,34]. H_2_O_2_ also interacts with hormones to control plant stress reactions and developmental processes. It may be easily detected in plants using different methods since it is the most stable ROS [35]. Therefore, together with malondialdehyde (MDA), H_2_O_2_ is one of the most frequently used oxidative stress markers in plants [36,37,38]. MDA is a product of lipid peroxidation, which includes oxidative damage of the fatty acids of cell membranes, lipoproteins, and other lipid-containing structures [39].

To combat the harmful effects of ROS, cells have evolved an effective, intricate system of enzymatic and non-enzymatic antioxidant defences. The latter category includes ascorbic acid, glutathione, flavonoids, phenols, and carotenoids [40]. These metabolites can significantly lower ROS and prevent cell damage, complementing the enzymatic antioxidant systems [41]. Together with photosynthetic pigments and osmolytes, they have been successfully used as drought stress biomarkers [10,18,42,43]. The build-up of osmolytes (or compatible solutes) in the cytosol is a typical response in plants to compensate for osmotic imbalance. Their accumulation helps stress-tolerant plants reduce cellular dehydration brought on by various abiotic stressors, such as salinity and drought. Osmolytes perform a variety of roles in stress tolerance in addition to their function in osmoregulation, such as operating as low-molecular-weight chaperones, ROS scavengers, or signalling molecules [44,45,46]. Proline is one of the most prevalent osmolytes in plants, which besides its role in osmoregulation serves as a signalling molecule and antioxidant, quenching O_2_, H_2_O_2_, and OH, stabilising ROS-scavenging, and maintaining a low NADPH (nicotinamide adenine dinucleotide phosphate) level [47].

The present study integrates morphological characteristics with the aforementioned biochemical stress indicators to conduct a comparative analysis of the responses to water deficit of two ornamental species of the genus *Tropaeolum*, namely *Tropaeolum majus* and *T. minus*. The genus *Tropaeolum* L. (fam. Tropaeolaceae) is distributed in Central and South America [48] and includes several worldwide popular ornamentals. *Tropaeolum majus* L. is a cultigen not known from the wild and probably originated in Peru by unintentional hybridisation between *T. minus* L. and *T. ferreyrae* Sparre [49]. Due to its large and brightly coloured flowers, it was introduced and became popular as a garden species or used in landscaping in North and Central America, Africa, Asia, Europe, Australia, New Zealand and the Oceanic Islands [50]. It is also used as an edible and medicinal plant [51,52,53] and is effective as an aphid trap in vegetable gardens [54]. The species often eludes cultivation through garden debris and is reported as invasive from different regions, such as the Canary Islands, Malta, Hawaii and California. As a climber, attaining a length up to 1 m or more [51], it outcompetes native vegetation by shading and smothering [55].

*Tropaeolum minus* L. was the first species of this genus cultivated in Europe, but gradually its popularity declined in favour of the more robust and showier *T. majus* [56]. It is a uniform species, with few cultivars, originating in Peru and Ecuador and cultivated throughout the world, not reported as naturalised or invasive [56]. Besides its interest as ornamental, it is beneficial for horticultural crops or fruit trees by deterring pests [56].

The objectives of this study were to assess which of these two species is more suitable to be grown in conditions of limited hydric resources and also to identify biochemical water stress markers helpful to characterise their mechanisms of response to drought. The plants were grown under controlled conditions and two levels of water deficit, and several morphological and biochemical parameters were determined in the harvested material. The relative resistance of the two species to water stress was assessed based on the multivariate statistical analysis of the obtained data.

## 2. Materials and Methods

### 2.1. Plant Material

Seeds were purchased from commercial suppliers (Vilmorin Seed Generation, Paris, France). The seeds were germinated for twenty days in standard Petri dishes (⌀ = 85 mm) in a growth chamber under controlled conditions, 12 h light/12 h dark, at 25 °C. Three weeks after germination, seedlings were individually placed in 12 cm diameter pots filled with a mixture of commercial peat and perlite (3:1), watered regularly with tap water, and transferred to a greenhouse at the Polytechnical University of Valencia, Spain, with natural light, 60% relative humidity and a temperature range of 17–23 °C.

### 2.2. Drought Treatments and Growth Parameters

Two months after transplanting the seedlings, when plants were fully-grown in the vegetative stage, stress treatments were initiated. Six replicates per treatment and species were placed in plastic trays (12 pots per tray) and watered twice weekly with tap water added to the trays. Plants in the control group received 1.5 L of tap water per irrigation, those in the intermediate water stress (IWS) group received 0.75 L, and those in the severe water stress (SWS) group did not receive any water at all. Substrate moisture expressed as volume percentages and soil electroconductivity (EC) expressed in dS m^−1^ were determined weekly, from the beginning to the end of the treatments, with a WET-2 Sensor (Delta-T Devices, Cambridge, UK).

After 30 days of treatment, when the soil moisture of the SWS treatment dropped to ca. 5% volumetric water content, the plant material was sampled. The roots were cleaned with a brush after separating the aerial portion, and both the above and below-ground parts were weighed and measured individually.

Morphological growth parameters such as the number of leaves, leaves fresh weight, dry weight and water content, stem length, fresh weight, total dry weight and water content, and root length, fresh weight, dry weight and water content were measured for all individual plants. Water content was determined by weighing a part of the harvested material before (fresh weight, FW) and after drying at 65 °C for 72 h (dry weight, DW), using the equation [(FW − DW)/FW] × 100.

Fresh plant material samples (0.05–0.1 g) were placed in adequately labelled 2 mL Eppendorf tubes and stored at −75 °C before being used for the biochemical tests. Dry material samples were kept in paper bags at room temperature.

### 2.3. Biochemical Analyses

The non-destructive measurements of chlorophyll, flavonols and anthocyanins contents, and the nitrogen balance index (NBI) were performed using the optical sensor Dualex Scientific^®^ (Force-A, Orsay, France). Dualex (dual excitation) is a field-portable device for measuring the UV absorbance of the leaf epidermis using two-fold excitation of chlorophyll fluorescence, which allows rapid measurements on attached leaves even in outdoor settings [57]. Measurements were performed on the adaxial and abaxial sides of three leaves from the top of each plant.

For quantitative estimation of photosynthetic pigments, fresh leaf material (0.05 g) was ground and extracted in 1 mL ice-cold 80% acetone (*v*/*v*). Extraction was carried out overnight on a shaker in the dark. Samples were centrifuged at 13,300× *g* for 10 min at 4 °C, and the supernatant was collected. The absorbance of the supernatant liquid was determined at 470 nm, 646 nm and 663 nm. The concentrations of chlorophyll *a* (Chl a), chlorophyll *b* (Chl b) and carotenoids (Caro) were calculated according to the classical Lichtenthaler and Wellburn method [58] and expressed in mg g^−1^ DW.

Two types of osmolytes were analysed in the study, proline (Pro) and total soluble sugars (TSS). Quantification of Pro in the leaves of the harvested plants was performed according to Bates et al. [59]. Fresh ground material (0.05 g) was extracted in 3% (*w*/*v*) aqueous sulphosalicylic acid and mixed with acid ninhydrin. Samples were then incubated in a water bath for 1 h at 98 °C, cooled on ice for 10 min, and extracted with toluene. The absorbance of the organic phase was measured spectrophotometrically at 520 nm. The standard curve was obtained with solutions containing known Pro concentrations, assayed in parallel. Pro contents were expressed as µmol g^−1^ DW.

Total soluble sugars were determined following the method of Dubois et al. [60]. Freshly ground leaf samples (0.05 g each) were extracted overnight with 80% methanol (*v*/*v*). After centrifugation at 13,300× *g* at 4 °C for 10 min, 500 µL of 5% phenol (*v*/*v*) and 2.5 mL of concentrated sulphuric acid were added to the supernatant to induce an exothermic reaction to caramelise the extracted sugar contents. Absorbance was measured at 490 nm. TSS contents were expressed as equivalents of glucose, the sugar used as the standard (mg eq. glucose g^−1^ DW).

Oxidative stress marker malondialdehyde (MDA), antioxidant compounds total phenolic compounds (TPC), and total flavonoids (TF) were measured in extracts prepared in 80% (*v*/*v*) methanol from 0.05 g ground fresh leaf material. For peroxide quantification (H_2_O_2_ measurements), samples (0.05 g) were homogenised in 500 µL of 0.1% trichloroacetic acid (*w*/*v*).

MDA quantification was performed according to Hodges et al. [61], with some modifications [62]. The extracts were mixed with 0.5% (*w*/*v*) thiobarbituric acid (TBA) prepared in 20% (*w*/*v*) trichloroacetic acid (TCA), or with 20% TCA without TBA for the corresponding controls. Samples were incubated in a dry block thermostat at 95 °C for 15 min, cooled on ice and centrifuged at 13,300× *g* for 10 min at 4 °C. MDA contents in the extracts were determined according to Taulavuori et al. [62], based on the extinction coefficient of the MDA-TBA adduct at 532 nm (155 mM^−1^ cm^−1^), after subtracting the non-specific absorbance at 440 and 600 nm. Finally, MDA concentrations were calculated in nmol g^−1^ DW.

Hydrogen peroxide (H_2_O_2_), another oxidative stress marker, was measured according to Loreto and Velikova [63]. Fresh leaf material (0.05 g) was extracted with a 0.1% (*w*/*v*) trichloroacetic acid (TCA) solution, and the homogenate was centrifuged at 13,300× *g* for 15 min at 4 °C. An aliquot of 500 µL supernatant was mixed with 500 µL of 10 mM potassium phosphate buffer (pH 7) and 1 mL of 1 M potassium iodide. The absorbance was determined at 390 nm, and a standard curve was obtained from samples containing known H_2_O_2_ concentrations, assayed in parallel. H_2_O_2_ concentrations were expressed as µmol g^−1^ DW.

The concentration of total phenolic compounds (TPC) was measured according to Blainski et al. [64], based on the reaction of the methanol extract with the Folin–Ciocalteu reagent in the presence of sodium carbonate. The samples were incubated at room temperature in darkness for 90 min, and the absorbance was measured at 765 nm. Samples with known gallic acid (GA) concentrations were assayed in parallel to obtain a calibration curve, and TPC contents were calculated as mg eq. GA g^−1^ DW.

Total flavonoids (TF) were quantified according to the protocol of Zhishen et al. [65]. The methanol extract of each sample was incubated with sodium nitrate for the nitration of aromatic rings containing a catechol group, followed by a reaction with AlCl_3_ at basic pH. The samples were quantified spectrophotometrically at 510 nm, and TF concentrations were expressed as equivalents of catechin (mg eq. C g^−1^ DW), which was utilised as the standard.

### 2.4. Statistical Analysis

One-way ANOVA was performed separately in each species to assess the impact of the water stress treatments (CON, IWS, and SWS) on *T. majus* and *T. minus*. Subsequently, the Tukey Honestly Significant Difference (HSD) post hoc test was applied to identify statistically significant differences between the treatments’ mean values, with a significance level of *p* < 0.05.

Two-way ANOVA was applied to check also the effect of species, in addition to that of the treatment, and the significance of the interaction of the two factors.

To gain a comprehensive understanding of the collected data and identify the primary drivers of variation, two types of multivariate descriptive statistics were employed: correlation analysis and principal component analysis (PCA). To improve the visual presentation of the results and avoid overlapping labels, we replaced the individual fresh and dry weights of root, stem, and leaf with their respective sum, obtaining the total fresh weight (TFW) and total dry weight (TDW). Similarly, the two components of chlorophyll, Chl a and Chl b, were replaced with their sum (Chl tot).

Two separate Pearson correlation matrices were calculated for *T. majus* and *T. minus* data, using a 95% confidence interval.

Two customised scatter plots were generated from the complete dataset to examine further the relationship between the chlorophyll and flavonoid levels measured with the spectrophotometer in the laboratory and those obtained through Dualex sensor measurements. The Pearson correlation coefficients (R) and their corresponding statistical significance levels (*p*-values) are shown in the plots.

For the PCA, all the measured traits were set as active quantitative variables, whereas the two species (*T. minus* and *T. majus*) and the three water stress treatments (CON, INS, SWS) were used as supplementary categorical variables that were not included in the principal components (PCs) determination. The PCs were computed by centring and scaling the quantitative variables, diagonalising the correlation matrix, and extracting the related eigenvectors and eigenvalues. The *p*-values of the correlation coefficients between the traits and the first two PCA components are provided in Table S1 of the Supplementary Materials. The eigen analysis is displayed in Figure S1 of the Supplementary Materials.

The analysis of variance and post hoc test were performed with the stats [66], Emmeans [67] and Multcomp [68] packages of the R 4.2.2 statistical software and were visualized with the ggplot2 [69] package. Additionally, the Corrr [70], Corrplot [71], and FactoMineR [72] packages were used for the multivariate analysis.

## 3. Results

### 3.1. Effects of Water Stress on Plant Growth

After four weeks of water stress treatment, when the substrate moisture was below 5% under severe water stress conditions, all plants were sampled, and their growth parameters were analysed. The two-way ANOVA indicated a significant effect of the treatment for different parameters but only the lengths of root and leaves varied significantly between species. The interaction of the two factors was significant only for the water content of roots and leaves (Table 1).

**Figure 1:**
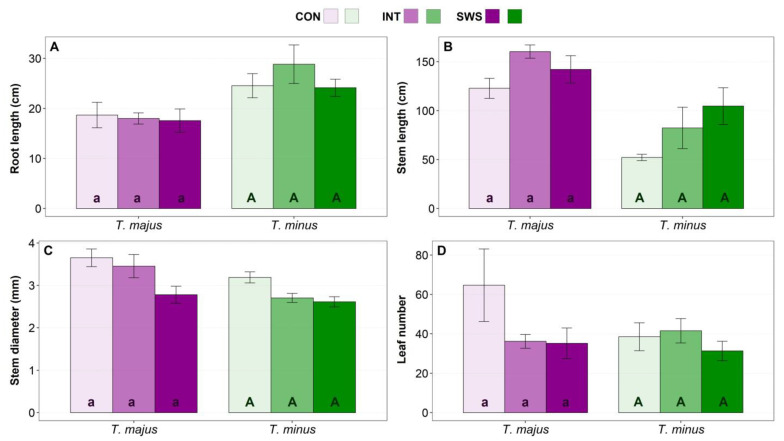
Growth parameters: root length (**A**), stem length (**B**), stem diameter (**C**) and leaf number (**D**) in *Tropaeolum majus* and *T. minus* plants grown under controlled conditions in the greenhouse, after 30 days of water stress treatment (CON—control, IWS—intermediate water stress, SWS—severe water stress). Values shown are means ± SE (*n* = 6). The same lowercase and uppercase letters indicate homogeneous groups between treatments for *T. majus* and for *T. minus*, respectively, according to the Tukey test (*p* ≤ 0.05).

The root length of *T. majus* plants did not vary significantly, with measurements ranging between 17.6 (SWS) and 18.7 cm (CON). In *T. minus,* only a slight, non-significant increase in the root length in the IWS treatment with a mean value of 28.8 cm was observed, as compared to the CON (24.1 cm) and SWS treatments (24.6 cm) (Figure 1A). Although increased root growth is a typical response of plants under water stress conditions for exploring moister layers of the soil when growing in pots, root expansion is limited.

The average stem length values slightly increased under stress treatments in the two species, but these variations were not statistically significant (Figure 1B). Similarly, the stem diameter was not significantly affected by the water stress treatments, although a slight decrease in the mean values was noticed in both species, in parallel to the increasing water deficit (Figure 1C).

A notable decline in the mean leaf number of about 50% was recorded in plants of *T. majus* under water stress conditions (IWS and SWS treatments), whereas for *T. minus,* only a minor decrease was observed in the SWS treatment. However, the differences with the corresponding control values were not significant for both species. Notably, the number of leaves in *T. minus* CON plants (~39) was much lower than in *T. majus* CON plants (~65) (Figure 1D). To summarise the results shown in Figure 1, a trend to increase the mean values of stem length and to decrease those of stem diameter and leaf number in response to water stress was observed in both studied species. Therefore, although slightly taller, the water-stressed plants were less vigorous than those in control, as their stem diameter and the number of leaves decreased. However, due to the variability in the measurements, reflected in relatively large SE values, the differences with the control, non-stressed plants were not statistically significant.

**Figure 2:**
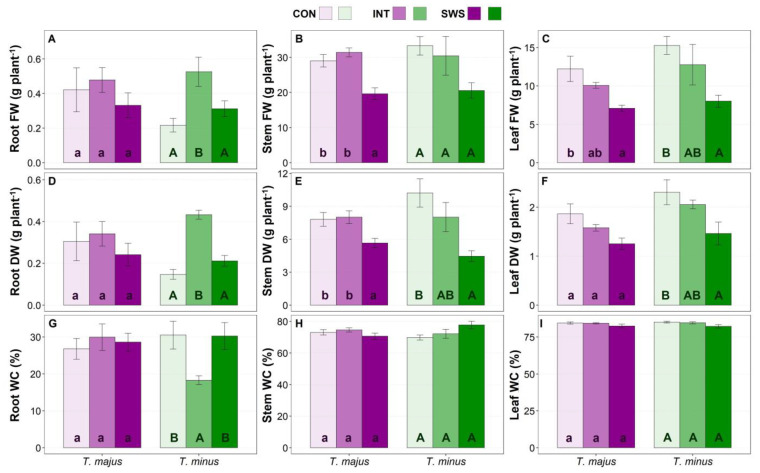
Weight and water content parameters: fresh weight (FW) of roots (**A**), stems (**B**), and leaves (**C**); dry weight (DW) of roots (**D**), stems (**E**) and leaves (**F**); water content (WC) of roots (**G**), stems (**H**) and leaves (**I**) in *Tropaeolum majus* and *T. minus* plants grown under controlled conditions in the greenhouse, after 30 days of water stress treatment (CON—control, IWS—intermediate water stress, SWS—severe water stress). Values shown are means ± SE (*n* = 6). The same lowercase and uppercase letters indicate homogeneous groups between treatments for *T. majus* and for *T. minus*, respectively, according to the Tukey test (*p* ≤ 0.05).

After recording the morphological parameters in the initial experimental phase, the fresh weight (FW), dry weight (DW), and water content (WC) of the roots, stems, and leaves were determined in control and water-stressed plants (Figure 2).

Water stress (IWS and SWS treatments) did not affect the growth of *T. majus* plants at the root level, as no significant changes compared to the control were detected in the root FW (Figure 2A), DW (Figure 2D) or WC (Figure 2G), as previously observed for root length (Figure 1A). The same results were obtained for *T. minus* plants subjected to severe water stress. However, in this species, root FW increased significantly, ca. 2.5-fold higher than in CON plants, in response to the IWS treatment (Figure 2A); the relative increase in root DW was even higher, about 3-fold (Figure 2D), partly due to the parallel decrease in root WC (Figure 2G).

The stem FW (Figure 2B) and DW (Figure 2E) of *T. majus* plants were not affected by the IWS treatment but decreased significantly under severe water stress (SWS) conditions, down to ca. 70% of the mean values measured for both parameters in the unstressed plants (CON). Mean FW (Figure 2B) and DW (Figure 2E) values of *T. minus* plants showed a decreasing trend with increasing water deficit intensity, although the differences with the control plants were statistically significant only for DW under severe water stress conditions (SWS treatment). Regarding the responses to the water stress treatments in terms of leaf FW (Figure 2C), both species showed a similar trend, with a reduction of mean FW values with increasing intensity of the applied treatment; however, the differences with the unstressed plants were significant only for the SWS treatment, amounting to ca. 42% and 48% of the corresponding controls for *T. majus* and *T. minus*, respectively. It is worth noting that leaf FW was higher in *T. minus* than in *T. majus* under all tested conditions. The same qualitative pattern of variation was also observed for the leaf DW measurements in both species, although in this case, the differences between mean values were significant only in *T. minus* plants (Figure 2F).

The two species showed high resistance to drought-induced dehydration of the aerial part of the plants, as stem (Figure 2H) and leaf (Figure 2I) WC did not change in response to the IWS or SWS treatments. A summary of all growth parameters represented as variation calculated in percentage in relation to their respective control value is presented in Supplementary Table S2.

### 3.2. Effect of Water Stress on Parameters Measured in the Greenhouse with an Optical Sensor

The differences in chlorophyll, flavonols and anthocyanins contents, and the nitrogen balance index (NBI), measured with a Dualex optical sensor, were analysed at the end of the water stress treatments (Table 2). No significant differences were observed in *T. majus* plants in any of the measured variables when comparing the different treatments. In *T. minus*, flavonol contents showed a significant reduction of 36% with respect to the control plants under severe water stress conditions. The water deficit also caused a progressive increase in the NBI of 12% (IWS) and 32% (SWS), although only the latter value significantly differed from the control. A summary of the optical sensor measurements represented as variation calculated in percentage in relation to their respective control value is presented in Supplementary Table S3.

The parameters quantified in the greenhouse showed only a small variation between species, but the interactions detected by the two-way ANOVA according to the two factors (species and treatment) were not significant (Table 3).

### 3.3. Effect of Water Stress on Biochemical Parameters

**Figure 3:**
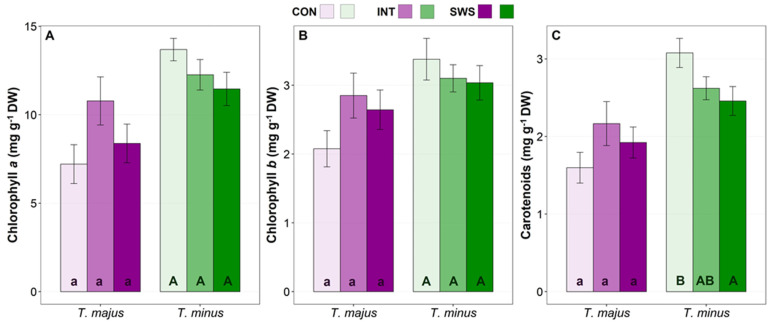
Effect of 30 days of water stress treatment (CON—control, IWS—intermediate water stress, SWS—severe water stress) on the contents of photosynthetic pigments in *T. majus* and *T. minus* leaves. Chlorophyll *a* (**A**), Chlorophyll *b* (**B**), and total carotenoids (**C**). Values shown are means ± SE (*n* = 6). The same lowercase and uppercase letters indicate homogeneous groups between treatments for *T. majus* and for *T. minus*, respectively, according to the Tukey test (*p* ≤ 0.05).

Although the concentrations of the biochemical compounds analysed showed generally significant differences between species, the interaction of the factors Species and Treatment detected by the two-way ANOVA were significant only for carotenoids, proline and hydrogen peroxide, indicating a similar trend in the two species’ responses to stress (Table 3). The concentration of the photosynthetic pigments was measured spectrophotometrically in the sampled leaves (Figure 3). The concentration of chlorophyll *a* (Figure 3A) and chlorophyll *b* (Figure 3B) did not vary significantly in response to water stress in any of the two analysed species. However, mean chlorophyll values showed opposite trends, increasing with respect to the control in *T. majus*, particularly in the IWS treatment, and decreasing progressively in *T. minus*. Carotenoid contents showed the same trend as chlorophylls in both species, although the reduction observed in *T. minus* was significant under SWS conditions (Figure 3C).

**Figure 4:**
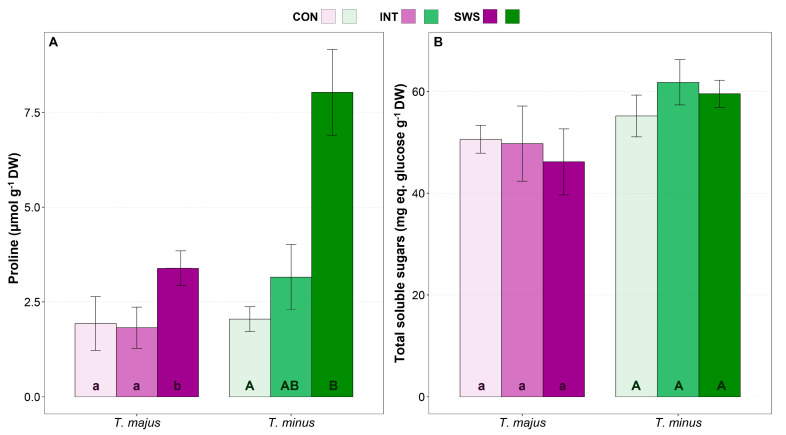
Effect of 30 days of water stress treatment (CON—control, IWS—intermediate water stress, SWS—severe water stress) on leaf concentrations of Proline (Pro) (**A**) and total soluble sugars (TSS) (**B**) in *T. majus* and *T. minus* plants. Values shown are means ± SE (*n* = 6). The same lowercase and uppercase letters indicate homogeneous groups between treatments for *T. majus* and for *T. minus*, respectively, according to the Tukey test (*p* ≤ 0.05).

During the water stress period, mean proline (Pro) concentration increased significantly in both species in response to the SWS treatment (Figure 4A). Leaf Pro contents were similar (ca. 2 µmol g^−1^ DW) in unstressed plants of both species, but the relative increase over control values was much higher in *T. minus*, about 4-fold, than in *T. majus* (1.75-fold). In any case, even the maximum absolute Pro concentration reached, ~8 µmol g^−1^ DW, was too low to have any relevant osmotic effect. On the other hand, no significant stress-induced changes were observed in total soluble sugars (TSS) contents in any of the two species (Figure 4B).

**Figure 5:**
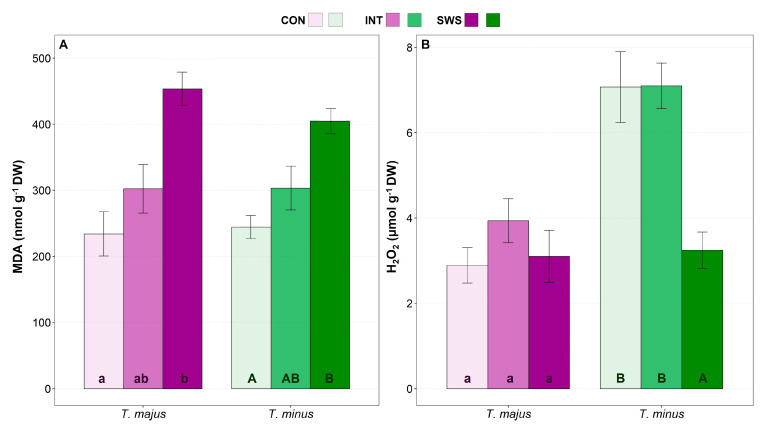
Effect of 30 days of water stress treatment (CON—control, IWS—intermediate water stress, SWS—severe water stress) on leaf concentrations of oxidative stress markers in *T. majus* and *T. minus* plants. Malondialdehyde (MDA) (**A**) and hydrogen peroxide (H_2_O_2_) (**B**). Values shown are means ± SE (*n* = 6). The same lowercase and uppercase letters indicate homogeneous groups between treatments for *T. majus* and for *T. minus*, respectively, according to the Tukey test (*p* ≤ 0.05).

Malondialdehyde (MDA) and hydrogen peroxide (H_2_O_2_) leaf concentrations in the *Tropaeolum* plants were measured to assess the potential drought-induced generation of secondary oxidative stress (Figure 5). MDA levels increased in parallel to the intensity of water stress, reaching values significantly higher than the controls under SWS conditions. The relative rise in MDA contents was more accentuated in *T. majus* than in *T. minus*, about 2-fold vs 1.5-fold, respectively, with respect to the non-stressed plants (Figure 5A). On the other hand, H_2_O_2_ contents showed a significant reduction of 54% in *T. minus* plants subjected to the SWS treatment, whereas they did not vary in *T. majus* (Figure 5B).

**Figure 6:**
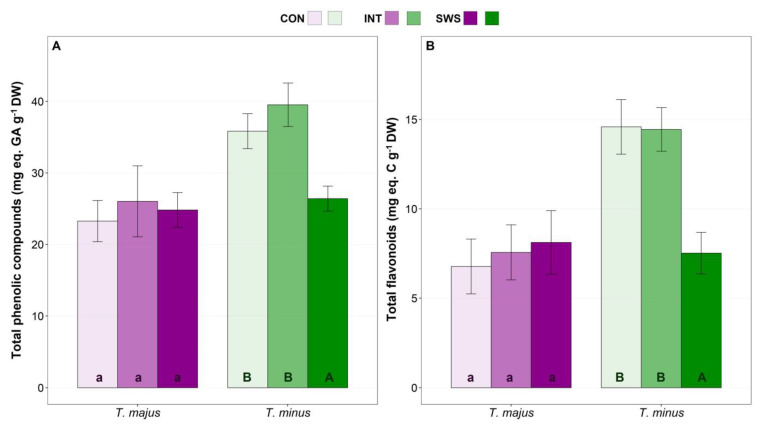
Effect of 30 days of water stress treatment (CON—control, IWS—intermediate water stress, SWS—severe water stress) on leaf concentrations of total phenolic compounds (TPC) (**A**) and total flavonoids (TF) (**B**) in *T. majus* and *T. minus* plants. Values shown are means ± SE (*n* = 6). The same lowercase and uppercase letters indicate homogeneous groups between treatments for *T. majus* and for *T. minus*, respectively, according to the Tukey test (*p* ≤ 0.05).

Significant changes in total phenolic compounds (TPC, Figure 6A) and total flavonoids (TF, Figure 6B) contents under water deficit conditions were registered only in *T. minus* plants. Specifically, compared to the corresponding non-stressed controls, leaf TPC and TF levels decreased by ca. 30% and 50%, respectively, under severe water stress. In the case of *T. majus* plants, no significant variation was observed in any of the treatments (Figure 6). A summary of all biochemical parameters represented as variation calculated in percentage in relation to their respective control value is presented in Supplementary Table S4.

### 3.4. Multivariate Analysis of the Results

**Figure 7:**
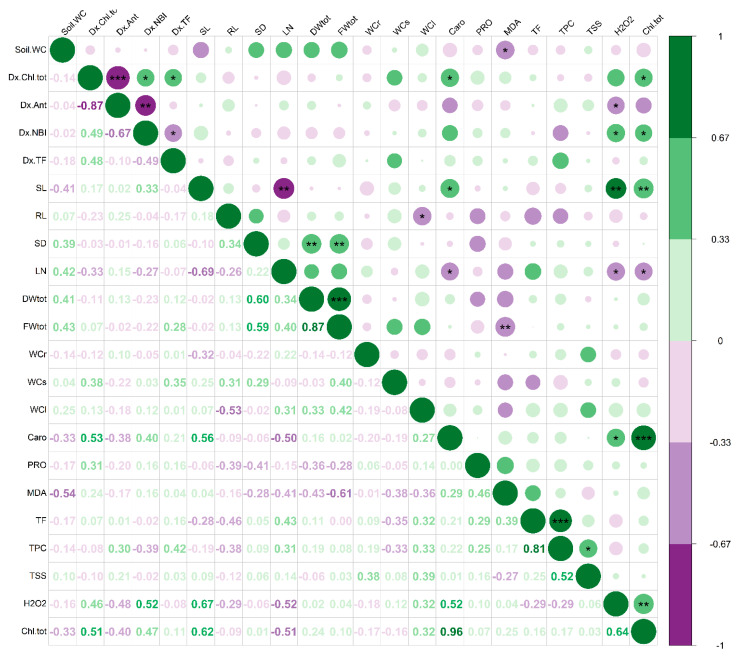
Matrix of the Pearson correlation coefficients between the 22 traits measured on *T. majus*. The strength of each correlation is represented by a circle above the diagonal, with larger and more intense circles indicating stronger correlations. Green circles indicate a positive correlation (from 0 to 1), whereas violet circles indicate a negative correlation (from 0 to −1). Significance levels are denoted by asterisks (*, **, and ***, at *p* < 0.05, 0.01, and 0.001, respectively). The numerical values of the correlation coefficients are displayed below the diagonal. Abbreviations: soil water content (soil WC), Dualex total chlorophyll (Dx Chl tot), Dualex anthocyanins (Dx Ant), Dualex nitrogen balance index (NBI), Dualex flavonols (Dx TF), stem length (SL), root length (RL), stem diameter (SD), leaf number (LN), total dry weight (DWtot), total fresh weight (FWtot), root water content (WCr), stem water content (WCs), leaf water content (WCl), carotenoids (Caro), proline (PRO), malondialdehyde (MDA), total phenolic compounds (TPC), total flavonoids (TF), total soluble sugars (TSS), hydrogen peroxide (H_2_O_2_), total chlorophyll (Chl tot).

**Figure 8:**
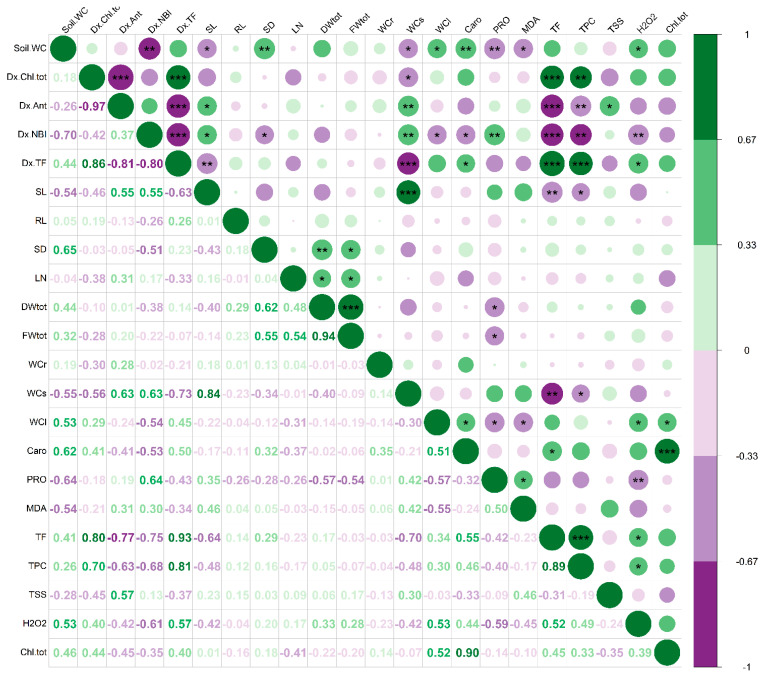
Matrix of the Pearson correlation coefficients between the 22 traits measured on *T. minus*. The strength of each correlation is represented by a circle above the diagonal, with larger and more intense circles indicating stronger correlations. Green circles indicate a positive correlation (from 0 to 1), whereas violet circles indicate a negative correlation (from 0 to −1). Significance levels are denoted by asterisks (*, **, and ***, at *p* < 0.05, 0.01, and 0.001, respectively). The numerical values of the correlation coefficients are displayed below the diagonal. Abbreviations as in Figure 7.

By analysing the *T. majus* and *T. minus* correlation matrices (Figure 7 and Figure 8), we could determine the magnitude and direction of the linear relationship between the morphological and biochemical traits. This analysis enabled us to identify the most effective biochemical markers for characterising and distinguishing the responses to drought stress in these two closely related species.

In *T. majus*, the total plant fresh weight exhibited a negative correlation with MDA content, highlighting that oxidative stress caused a reduction in plant growth. Notably, the total chlorophyll content showed a negative correlation with the leaf number, suggesting that reduced leaf production led to higher pigment concentration in the photosynthetic organs of this species. In addition, the leaf number correlated negatively with stem length, indicating that the plant may reduce leaf production to minimise water loss through transpiration while increasing stem length to reach higher or more favourable locations for light exposure or airflow. These changes can help maintain optimal growth under drought stress conditions.

In *T. minus*, the total plant fresh and dry weight negatively correlated with the proline levels (Figure 8). Indeed, proline was the most accumulated osmoregulatory compound in this species in response to drought stress. However, its osmoprotective and antioxidant contribution was insufficient to prevent the generation of reactive oxidative species, as evidenced by the positive correlation found between proline and MDA. MDA, in turn, showed a negative correlation with the leaf water content, indicating that oxidative stress compromised the cellular membranes and their ability to maintain cellular homeostasis.

It is worth noting that in both species, the content of H_2_O_2_, a reactive oxygen species generated in response to a variety of stress conditions, was positively correlated with pigments (carotenoids and total chlorophyll) contents, flavonoid and phenols, as well as with the leaf water content.

**Figure 9:**
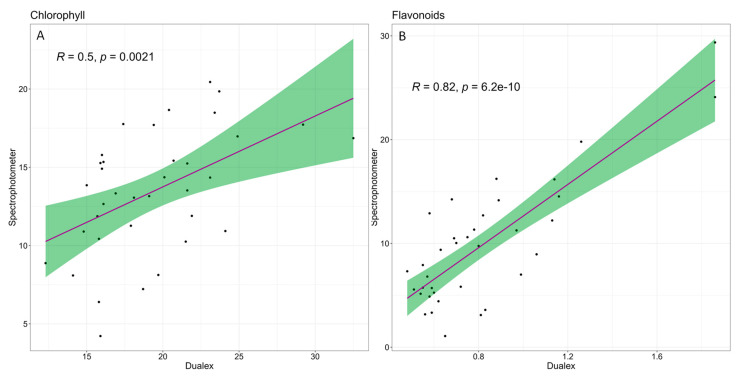
Scatterplot between (**A**) the content of chlorophyll measured with the Dualex sensor and the spectrophotometer and (**B**) the content of flavonoids measured with the Dualex sensor and the spectrophotometer. The green shaded area around each line represents a 95% confidence interval for the relationship between the variables.

Several parameters were measured with both the optical sensor and spectrophotometry. The measurements of the chlorophyll content performed in the greenhouse with the Dualex sensor before plant harvest and by spectrophotometry after plant harvest on frozen leaf tissue showed a significant positive correlation (Figure 9A), with a Pearson correlation coefficient of 0.5, indicating a moderate positive association between the two sets of measurements. The correlation between the flavonoid content measured with the Dualex sensor and the spectrophotometer was even stronger, with a correlation index of 0.82 (Figure 9B), indicating a strong positive correlation between the two measurement methods.

**Figure 10:**
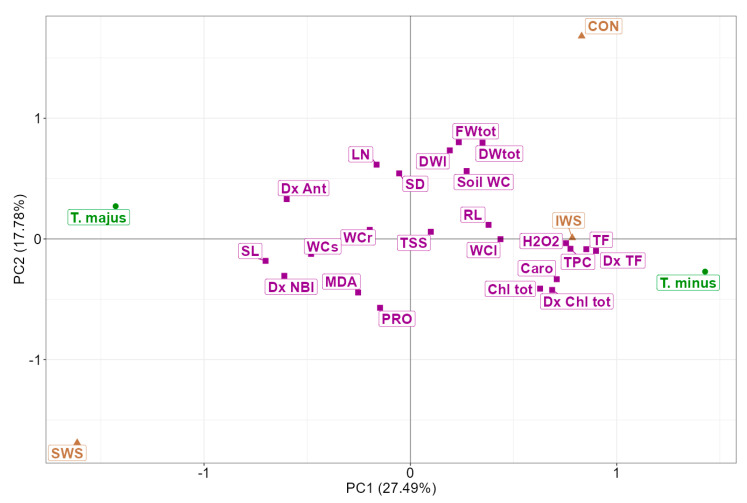
PCA biplot of the 22 measured traits. The brown triangles represent the water treatments: control (CON), intermediate water stress (IWS), and severe water stress (SWS). The green circles indicate the two investigated species (*T. minus* and *T. majus*), whereas the violet squares display the 22 measured traits. Abbreviations as in Figure 7.

To obtain an all-encompassing view of the observed data and identify which variables were the most important in explaining the variation in the gathered data, we performed a principal component analysis (PCA, Figure 10), a statistical technique used to reduce the dimensionality of the dataset while retaining as much of the original information as possible. The first principal component (PC1), which accounted for 27.5% of the total variability, provided a clear separation of the effects of the three treatments (CON, IWS and SWS) and allowed for the distinction of their relative impact on the two species. The barycenters of *T. minus* and the control and intermediate salt stress treatments were located on the positive side of the X-axis, showing a positive correlation with PC1. On the contrary, in *T. majus*, the severe salt stress treatments had barycenters on the negative side of the X-axis, indicating a negative correlation with PC1. This arrangement confirmed that *T. minus* performed better under control and intermediate water stress, whereas *T. majus* exhibited higher resistance under severe water stress. The second axis, which explained approximately 18% of the total variation, summarised the distinction between the two species regarding biochemical compounds. *T. minus*, with higher levels of proline, flavonoids, and pigments, was located on the negative side of PC2 along with the barycenters of these biochemical markers, reflecting their higher content in this species, even in the absence of drought stress, except for proline. The contribution of the total soluble sugar content was relatively insignificant, as indicated by its position close to the axis intersection, suggesting an overall negligible contribution to the plant’s response to drought stress. The root, stem and leaf water contents also showed minimal contribution in elucidating the data variation and differentiating the response of the two plant species under examination.

## 4. Discussion

Water stress in plants first becomes apparent in a reduction in growth due to interference with numerous physiological and biochemical processes. Drought inhibits the growth of plants more than any other environmental component by affecting photosynthesis, chlorophyll synthesis, nutrient metabolism, ion uptake and translocation, respiration, and carbohydrate metabolism [73,74]. Reduced leaf water potential and turgor pressure, stomatal closure, and reduced cell development and enlargement are the hallmarks of drought stress in plants [75]. The investigated species showed a general trend of growth reduction, but not for all analysed parameters. There are numerous reports on the reduction of stem length, diameter and leaf number under drought due to the inhibition of cell division, cell expansion, or both [76,77]. However, the two studied species showed only a non-significant variation in these parameters. Similarly, all traits related to roots (length, fresh and dry weight) did not differ significantly in the three treatments. On the contrary, water stress, especially the complete absence of irrigation, strongly affected the dry and fresh weight of stems and leaves in both species. Compared to control values, the reduction of the total fresh weight under severe water stress was similar in both species, about 35% in *T. majus* and 40% in *T. minus*. The differences between species were more accentuated when considering the reduction in total dry weight in the SWS treatment, 30% and over 50% of the control in *T. majus* and *T. minus*, respectively. Therefore, although several growth parameters varied similarly in both species in response to drought, *T. majus* is relatively more tolerant to severe water deficit than *T. minus*, as dry weight reduction is one of the most reliable indicators of plants’ susceptibility to water stress [78].

As stated earlier, physiological and biochemical parameters are helpful in screening for drought tolerance in ornamental plants [10,13,79]. Under severe water deficit, photosynthetic pigments usually show a decline in concentration [80,81,82,83,84], and maintaining their levels constant may indicate drought tolerance [85]. However, depending on the genotype and the drought stress duration, these compounds’ concentrations may not vary or even increase under drought [4,86,87]. In the present work, when measuring possible variations on photosynthetic pigments—chlorophylls and carotenoids—in response to the water stress treatments, the only significant change detected was the decrease in total carotenoids contents in *T. minus* plants subjected to the SWS treatment.

Proline, a “key player” in osmotic regulation, is an excellent indicator of stress, as its concentration usually increases under osmotic stress [88]. However, a higher concentration of this compatible solute is not always related to better stress tolerance, as many examples of comparative studies reported a higher proline accumulation in the most susceptible genotypes [89,90]. This also seems to be the case in the analysed *Tropaeolum* species since *T. minus*, less drought tolerant than *T. majus*, showed a higher absolute proline concentration and a stronger relative increase over control levels in the plants subjected to the SWS treatment. The multivariate analysis showed a negative correlation of proline with growth parameters in both species, but its concentrations were too low to be considered essential in their osmotic adjustment. Therefore, in *Tropaeolum* (or at least in the two species analysed in the present work), proline can be considered a suitable marker of water stress but is probably not directly involved in tolerance mechanisms based on osmotic adjustment under drought conditions.

Of all other biochemical parameters, MDA also showed a strong negative correlation with substrate moisture and plant growth parameters. MDA has been long reported as an ideal marker of lipid peroxidation during oxidative stress [91]. Although MDA reliability as an oxidative stress biomarker has been recently questioned due to the variability of reported data [92], numerous examples of significant correlations exist between its concentrations and the levels of oxidative stress suffered by plants [93,94]. In the two *Tropaeolum* species, concentrations of MDA increased gradually with the severity of water stress, reaching the highest levels in the plants subjected to the SWS treatment.

Another widely used indicator of oxidative stress is H_2_O_2_, a moderately reactive ROS, produced when the superoxide radical (O_2_^−^) is dismutated into O_2_ and H_2_O_2_ [95]. However, in this study, H_2_O_2_ concentrations did not vary in *T. majus* and even decreased in *T. minus* but, in both species, its levels were positively correlated with the content of photosynthetic pigments, flavonoids and phenols, as well as with the leaf water content. Indeed, H_2_O_2_ also acts as a signalling molecule in response to stress, activating various pathways that lead to the upregulation of antioxidant defences, which help protect the plant against oxidative damage and to maintain cellular homeostasis. The higher content of H_2_O_2_ may have promoted the accumulation of flavonoids and phenolic compounds; the antioxidant activity of these metabolites could enhance the protection of the photosynthetic organ, contributing to the maintenance of the leaf water content and the preservation of the pigments pool.

As the multivariate analysis highlights, the results indicate similar responses of both species to drought but a more pronounced susceptibility to severe water stress in *T. minus*. The expansion of *T. majus* as a naturalised or even invasive species may be partially related to its ability to adapt better to conditions of soil water scarcity. This can be explained by its more efficient antioxidant system, as reflected by the concentrations of carotenoids, total phenolics and flavonoids, which did not vary in *T. majus* but suffered a reduction under severe water stress treatment in *T. minus*.

Finally, a practical aspect of this work is that, although values of chlorophyll and flavonols measured by Dualex and spectrophotometric assays differ, there is a good correlation in the pattern of variation registered by these two methods. Thus, simple and non-invasive measurements of these compounds by this optical sensor can be trustworthy in the species analysed.

## 5. Conclusions

The responses to water deficit of *Tropaeolum majus* and *T. minus* plants, evaluated by quantifying several growth and biochemical parameters, were similar. However, the smaller reduction of the total fresh and dry weight of *T. majus*, indicated its relatively higher tolerance to severe water stress. Both species behaved similarly under mild water stress conditions, but the PCA revealed that *T. minus* performed better under control and intermediate water stress. Proline and malondialdehyde were identified as the optimal biomarkers of water stress in the two species. A positive correlation in the concentration of chlorophylls and flavonoids was found when analysed with the Dualex optical sensor in the greenhouse or by spectrophotometric assays in the laboratory, proving that Dualex can be used as a reliable indicator of the pattern of variations of these compounds.

## Figures and Tables

**Table 1:** Two-way ANOVA (F values) considering the effect of Species (S), Treatment (T), and their interactions (S × T) on growth parameters: stem length (SL), root length (RL), stem diameter (SD), leaf number (LN), fresh weight root (FWr), dry weight root (DWr), fresh weight stem (FWs), dry weight stem (DWs), fresh weight leaves (FWl), dry weight leaves (DWl), root water content (WCr), stem water content (WCs) and leaves water content (WCl).

Parameter	S	T	S × T
SL	29.956 ***	4.220 *	1.195 ^ns^
RL	14.740 ***	0.567 ^ns^	0.59 ^ns^
SD	9.452 **	7.779 **	1.261 ^ns^
LN	1.160 ^ns^	2.020 ^ns^	1.501 ^ns^
FWr	0.436 ^ns^	1.852 ^ns^	0.72 ^ns^
DWr	0.205 ^ns^	2.284 ^ns^	1.030 ^ns^
FWs	0.362 ^ns^	9.704 ***	0.419 ^ns^
DWs	0.317 ^ns^	11.034 ***	2.192 ^ns^
FWl	3.714 ^ns^	9.810 ***	0.322 ^ns^
DWl	2.827 ^ns^	3.572 *	0.14 ^ns^
WCr	0.706 ^ns^	1.765 ^ns^	3.731 *
WCs	0.077 ^ns^	0.997 ^ns^	3.983 *
WCl	0.12 ^ns^	4.781 *	0.108 ^ns^

*, **, *** significant at *p* = 0.05, 0.01 and 0.001, respectively; ns: not significant.

**Table 2:** Dualex parameters, chlorophyll concentration, leaf epidermal flavonols, anthocyanins and nitrogen balance index (NBI) as affected by 30 days of water stress treatments (CON—control, IWS—intermediate water stress, SWS—severe water stress).

Plant name	Treatment	Chlorophyll (µg cm^–2^)	Flavonols (µg cm^–2^)	Anthocyanins (µg cm^–2^)	Nitrogen Balance Index (NBI)
*T. majus*	CON	17.7 ± 1.0 a	0.7 ± 0.0 a	0.3 ± 0.0 a	27.6 ± 0.1 a
	IWS	19.5 ± 1.7 a	0.8 ± 0.0 a	0.3 ± 0.0 a	27.5 ± 0.1 a
	SWS	18.2 ± 2.0 a	0.7 ± 0.0 a	0.3 ± 0.0 a	28.8 ± 1.0 a
*T. minus*	CON	20.2 ± 1.0 A	1.1 ± 0.0 B	0.3 ± 0.0 A	19.5 ± 0.1 A
	IWS	22.2 ± 2.9 A	1.1 ± 0.0 B	0.3 ± 0.0 A	22.2 ± 0.3 AB
	SWS	18.7 ± 1.3 A	0.7 ± 0.0 A	0.3 ± 0.0 A	28.5 ± 0.0 B

Values shown are means ± SE (*n* = 6). The same lowercase and uppercase letters indicate homogeneous groups between treatments for *T. majus* and for *T. minus*, respectively, according to the Tukey test (*p* ≤ 0.05).

**Table 3:** Two-way ANOVA (F values) considering the effect of Species (S), Treatment (T), and their interactions (S × T) on Dualex optical sensor measurements [total chlorophyll (Dx Chl tot), anthocyanins (Dx Ant), nitrogen balance index (NBI), and flavonols (Dx TF)] and on biochemical parameters [chlorophyll *a* (Chl a), chlorophyll *b* (Chl b), carotenoids (Caro), proline (PRO), malondialdehyde (MDA), total phenolic compounds (TPC), total flavonoids (TF), total soluble sugars (TSS), and hydrogen peroxide (H_2_O_2_)].

Parameter	S	T	S × T
Dx Chl tot	1.703 ^ns^	0.997 ^ns^	0.243 ^ns^
Dx Ant	1.312 ^ns^	0.393 ^ns^	0.286 ^ns^
NBI	7.024 **	3.232 ^ns^	1.810 ^ns^
Dx TF	8.117 **	3.276 ^ns^	1.942 ^ns^
Chl a	19.405 ***	1.269 ^ns^	3.116 ^ns^
Chl b	8.334 **	0.407 ^ns^	2.168 ^ns^
Caro	24.302 ***	0.526 ^ns^	3.892 *
Pro	7.690 **	10.089 ***	3.405 *
MDA	0.108 ^ns^	8.585 ***	0.233 ^ns^
TF	6.644 *	1.233 ^ns^	2.134 ^ns^
TPC	6.520 *	1.320 ^ns^	1.124 ^ns^
TSS	5.290 *	0.198 ^ns^	0.396 ^ns^
H_2_O_2_	28.512 ***	9.185 ***	6.718 **

*, **, *** significant at *p* = 0.05, 0.01 and 0.001, respectively; ns: not significant.

## Data Availability

Data are contained within the article and Supplementary Material.

## Supplementary Materials

The following supporting information can be downloaded at: https://www.mdpi.com/article/10.3390/life13040960/s1, Figure S1: Eigen analysis of PCA; Table S1: Correlation coefficients between the first two PCs (PC1 and PC2) and the variables included in PCA. Abbreviations: Carotenoids (Caro), total chlorophyll (Chl tot), total dry weight (DWtot), Dualex anthocyanins (Dx Ant), Dualex total chlorophyll (Dx Chl tot), Dualex nitrogen balance index (NBI), Dualex flavonols (Dx TF), total fresh weight (FWtot), hydrogen peroxide (H_2_O_2_), leaf number (LN), malondialdehyde (MDA), proline (PRO), root length (RL), stem diameter (SD), stem length (SL), soil water content (soil WC), total flavonoids (TF), total phenolic compounds (TPC), total soluble sugars (TSS), leaf water content (WCl), root water content (WCr), stem water content (WCs), IWS—intermediate water stress, CON—control, SWS—severe water stress; Table S2: Variation of growth parameters such as stem length (vSL), root length (vRL), stem diameter (vSD), leaf number (vLN), fresh weight root (vFWr), dry weight root (vDWr), fresh weight stem (vFWs), dry weight stem (vDWs), fresh weight leaves (vFWl), dry weight leaves (vDWl), root water content (vWCr), stem water content (vWCs), leaves water content (vWCl) in intermediate water stress (IWS) and severe water stress (SWS) calculated in percentage in relation to their respective control (CON) value; Table S3: Variation of dualex optical sensor measurements such as total chlorophyll (vDx Chl tot), anthocyanins (vDx Ant), nitrogen balance index (vNBI), flavonols (vDx TF) in intermediate water stress (IWS) and severe water stress (SWS) calculated in percentage in relation to their respective control (CON) value; Table S4: Variation of biochemical parameters such as Chlorophyll *a* (vChl a), Chlorophyll *b* (vChl b), carotenoids (vCaro), proline (vPRO), malondialdehyde (vMDA), total phenolic compounds (vTPC), total flavonoids (vTF), total soluble sugars (vTSS), hydrogen peroxide (vH_2_O_2_) in intermediate water stress (IWS) and severe water stress (SWS) calculated in percentage in relation to their respective control (CON) value.
