# Supplementary material for: Evaluation of Drought Responses in Two Tropaeolum Species Used in Landscaping through Morphological and Biochemical Markers
Source: Life (Basel). 2023 Apr 6;13(4):960. doi: 10.3390/life13040960 (PMC10145515; doi:10.3390/life13040960)
Supplement: Supplementary file 1 [file life-13-00960-s001.zip › life-2321684-supplementary.pdf]

Table S1. Correlation coefficients between the first two PCs (PC1 and PC2) and the variables included in PCA. Abbreviations: Carotenoids (Caro), total chlorophyll (Chl tot), total dry weight (DWtot), dualex anthocyanins (Dx Ant), dualex total chlorophyll (Dx Chl tot), dualex nitrogen balance index (NBI), dualex flavonols (Dx TF), total fresh weight (FWtot), hydrogen peroxide (H_2O_2), leaf number (LN), malondialdehyde (MDA), proline (PRO), root length (RL), stem diameter (SD), stem length (SL), soil water content (soil WC), total flavonoids (TF), total phenolic compounds (TPC), total soluble sugars (TSS), leaf water content (WCl), root water content (WCr), stem water content (WCs), IWS - intermediate water stress, CON - control, SWS - severe water stress.

	PC1		PC2	
	R	*P*	R	*P*
Quantitative variables				
Caro	0.720	0.000	-0.344	0.040
Chl tot	0.644	0.000	-0.425	0.010
DWtot	0.305	0.070	0.723	0.000
Dx Ant	-0.612	0.000	0.305	0.070
Dx Chl tot	0.705	0.000	-0.386	0.020
Dx NBI	-0.599	0.000	-0.359	0.032
Dx TF	0.906	0.000	-0.032	0.855
FWtot	0.188	0.273	0.697	0.000
H_2O_2	0.753	0.000	-0.032	0.854
LN	-0.189	0.269	0.636	0.000
MDA	-0.239	0.161	-0.525	0.001
PRO	-0.128	0.456	-0.655	0.000
RL	0.367	0.028	0.051	0.769
SD	-0.074	0.668	0.584	0.000
SL	-0.689	0.000	-0.182	0.288
Soil WC	0.253	0.136	0.645	0.000
TF	0.858	0.000	-0.018	0.918
TPC	0.781	0.000	-0.026	0.882
TSS	0.093	0.591	0.015	0.932
WCl	0.447	0.006	0.128	0.457
WCr	-0.200	0.243	0.072	0.678
WCs	-0.481	0.003	-0.216	0.205
Categorical variables				
IWS	0.778	0.199	1.737	0.000
CON	0.747	0.218	-0.010	0.983
SWS	-1.524	0.009	-1.727	0.000
T. majus	-1.417	0.000	0.401	0.219
T. minus	1.417	0.000	-0.401	0.219

Table S2. Variation of growth parameters such as stem length (vSL), root length (vRL), stem diameter (vSD), leaf number (vLN), fresh weight root (vFWr), dry weight root (vDWr), fresh weight stem (vFWs), dry weight stem (vDWs), fresh weight leaves (vFWl), dry weight leaves (vDWl), root water content (vWCr), stem water content (vWCs), leaves water content (vWCl) in intermediate water stress (IWS) and severe water stress (SWS) calculated in percentage in relation to their respective control (CON) value.

Parameter	*T. majus*			*T. minus*		
	CON	IWS	SWS	CON	IWS	SWS
SL	100.00	130.53	115.74	100.00	157.83	200.64
RL	100.00	96.34	94.02	100.00	117.45	98.30
SD	100.00	94.61	76.13	100.00	84.79	81.91
LN	100.00	55.93	54.38	100.00	107.79	81.39
FWr	100.00	113.44	78.66	100.00	243.08	143.85
DWr	100.00	108.18	67.52	100.00	91.51	61.79
FWs	100.00	82.50	57.98	100.00	83.61	52.50
DWs	100.00	112.02	79.23	100.00	295.45	144.32
FWl	100.00	102.52	72.39	100.00	78.43	43.62
DWl	100.00	84.69	67.23	100.00	89.15	63.42
WCr	100.00	111.81	106.86	100.00	60.05	99.27
WCs	100.00	102.15	96.73	100.00	103.44	111.43
WCl	100.00	99.86	97.58	100.00	99.46	96.66

Table S3. Variation of dualex optical sensor measurements such as total chlorophyll (vDx Chl tot), anthocyanins (vDx Ant), nitrogen balance index (vNBI), flavonols (vDx TF) in intermediate water stress (IWS) and severe water stress (SWS) calculated in percentage in relation to their respective control (CON) value.

Parameter	*T. majus*			*T. minus*		
	CON	IWS	SWS	CON	IWS	SWS
Dx Chl tot	100.00	110.28	103.11	100.00	109.74	92.49
Dx Ant	100.00	101.75	102.34	100.00	96.10	112.34
NBI	100.00	99.58	104.29	100.00	113.93	146.24
Dx TF	100.00	116.24	100.77	100.00	106.45	62.11

Table S4. Variation of biochemical parameters such as Chlorophyll *a* (vChl a), Chlorophyll *b* (vChl b), carotenoids (vCaro), proline (vPRO), malondialdehyde (vMDA), total phenolic compounds (vTPC), total flavonoids (vTF), total soluble sugars (vTSS), hydrogen peroxide (vH_2O_2) in intermediate water stress (IWS) and severe water stress (SWS) calculated in percentage in relation to their respective control (CON) value.

Parameter	*T. majus*			*T. minus*		
	CON	IWS	SWS	CON	IWS	SWS
Chl a	100.00	149.55	116.25	100.00	89.59	83.74
Chl b	100.00	137.24	127.27	100.00	91.78	89.85
Caro	100.00	135.62	120.37	100.00	85.20	79.88
Pro	100.00	94.18	175.35	100.00	154.10	392.27
MDA	100.00	129.24	193.80	100.00	124.08	165.53
TF	100.00	111.60	119.83	100.00	99.00	51.56
TPC	100.00	111.88	106.69	100.00	110.30	73.68
TSS	100.00	98.34	91.22	100.00	111.96	107.91
H_2O_2	100.00	136.13	107.30	100.00	100.42	45.91

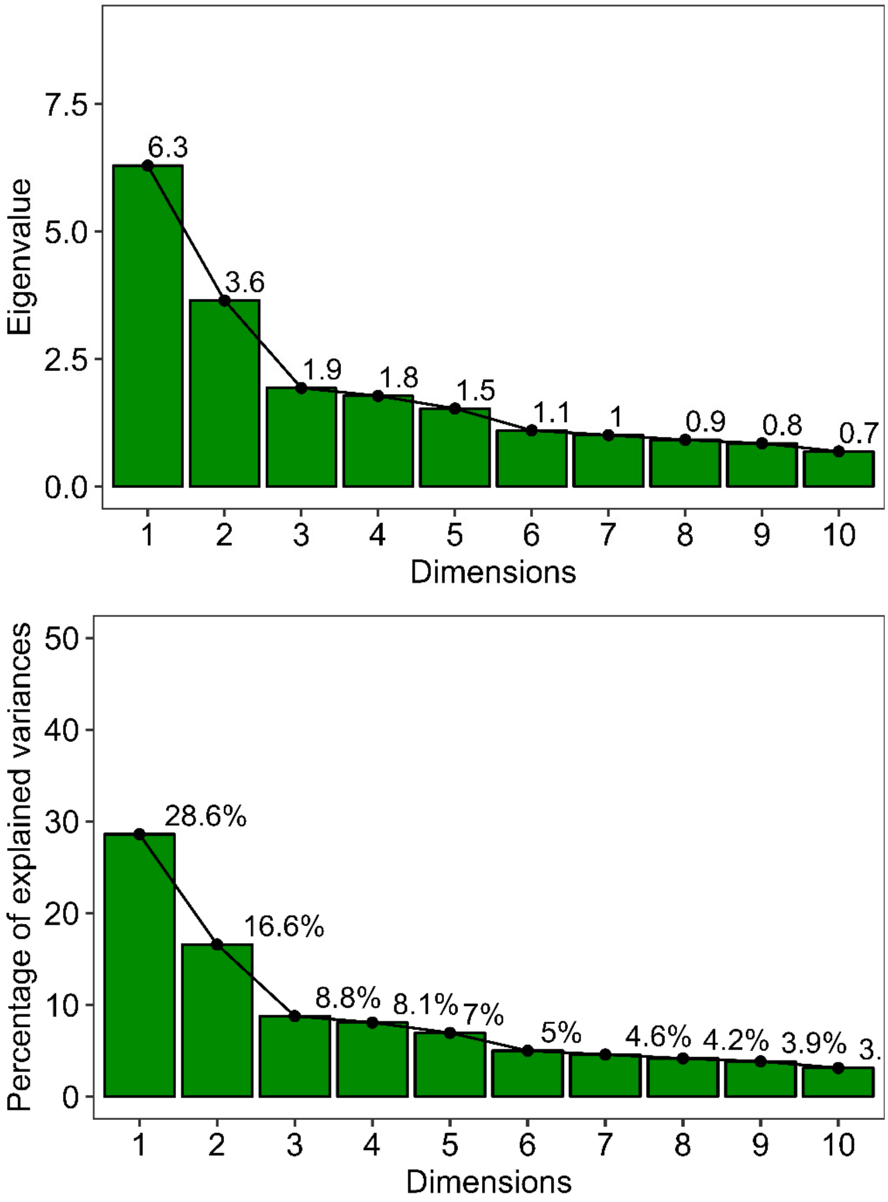

Figure S1. Eigen analysis of PCA
